# Historic review: select chapters of a history of stroke

**DOI:** 10.1186/s42466-020-00082-0

**Published:** 2020-12-01

**Authors:** Axel Karenberg

**Affiliations:** Institute for the History of Medicine and Medical Ethics, Medical Faculty, University Hospital Cologne, University of Cologne, Joseph-Stelzmann-Str. 20, 50931 Köln, Germany

**Keywords:** Stroke/history, Cerebrovascular disorders/history, Intracranial hemorrhages/history, Paralysis/history, Neurology/history, Stroke/pathology, Historical article

## Abstract

**Background:**

There is no shortage of books, chapters and papers on the history of stroke focusing predominantly on the last 150 years and enumerating endless “milestones”. Instead of adding another article to this body of knowledge, this essay aims at ensuring awareness for the “big picture”, the “grandes routes”, and the “striking breakes” without overloading the reader with too much detail.

**Results:**

From a medical point of view, the history of stroke consists of two periods: the early era from the beginnings to 1812, and the following period from 1812 up to the present. It is argued that both periods require different methodical approaches, including disparate historiographical perspectives and varying forms of interpretation. In order to fully understand medical writings of the Greco-Roman era (Hippocratic writings, Galenic corpus) on “apoplexy”, a solid knowledge of ancient doctrines concerning health and disease is indispensable. During the Middle Ages, the spiritual perspective can be highlighted by focusing on miracle healing and patron saints. While stroke basically remained a conundrum for many doctors and patients in early modern times (ca. 1500–1800; Platter, Wepfer), the revolutionary perception and definition of the disease as a result of a lesion in the 1810s (Rochoux, Rostan) opened the door to a productive relationship of the upcoming discipline “neurology” with the natural sciences during the nineteenth century and beyond (Virchow et al.). The mostly unwritten history of stroke in the twentieth century should not only include the medical, but also the patient’s and the societal perspective.

**Conclusion:**

A deeper insight into the recent and distant past will produce better educated strokologists – physicians who are able to put their own work into perspective.

## Background

“The true protagonists on the stage of medical history are the diseases”. Unfortunately, historical research often neglected this statement by French medical historian Charles Daremberg. We know a lot about famous physicians [1] and the social framing of medicine in the past [2], but comparatively little about the story of diseases themselves. Moreover, if and when they are studied by historians, the focus was and is usually put on epidemics and infectious diseases. 95% of the publications are on the history of plague, tuberculosis, syphilis and AIDS. In contrast, this essay argues that we can learn a lot about our medical past from non-epidemic, non-infectious illnesses. An excellent illustration of this approach is studying the history of stroke.

Stroke is today the second most common cause of death worldwide after heart disease, but before cancer [3]. In 2010, approximately 17 million people suffered a stroke, and another 33 million people have previously had a stroke and are still alive [4]. Obviously, we are really dealing with a “protagonist”. There seems to be just one open question to be resolved: how to assess appropriately the role of stroke in history?

### Historical models and perspectives

From a medical point of view, the history of stroke comprises two periods: A first era from the beginnings to 1812, and a second timeframe from 1812 to the present (see below). However, these different time intervals require different approaches by the historian.

The modern period (i.e., the 19th and 20th centuries) can convincingly be described using a model of medical progress, which can also be called the “embryonic knowledge approach” [5]. The scientific findings of one decade can be seen as the nucleus of knowledge to be discovered in the next decade, and so on and so forth. In this perspective, Virchow’s description of thrombosis and embolism of 1846 is somehow the basis for almost all knowledge about these conditions up to the end of the twentieth century. This story of progress was mainly played out in hospitals, dissection rooms and labs, and these institutions were and are predominantly located in parts of Europe, Australasia, and the US. Thus the historian dealing with this modern period needs only a working knowledge in few modern languages to read the primary sources, if, for instance, she or he wants to find out since when blood pressure and cerebral haemorrhage were connected or how the story of secondary prevention developed.

However, this model of progressing science is not a very suitable one for the pre-1800 period. For centuries, there were no advancements at all in a modern sense of the word, and if so, they were very slow. Hence a different concept is needed for that period, and an appropriate one is the “strange object approach” [5]. According to this approach, a contemporary physician has to admit that her/his present-day medical knowledge is of very little help to understand peculiar early teachings about stroke, because they differ so much from the current actual knowledge.

**Fig. 1:**
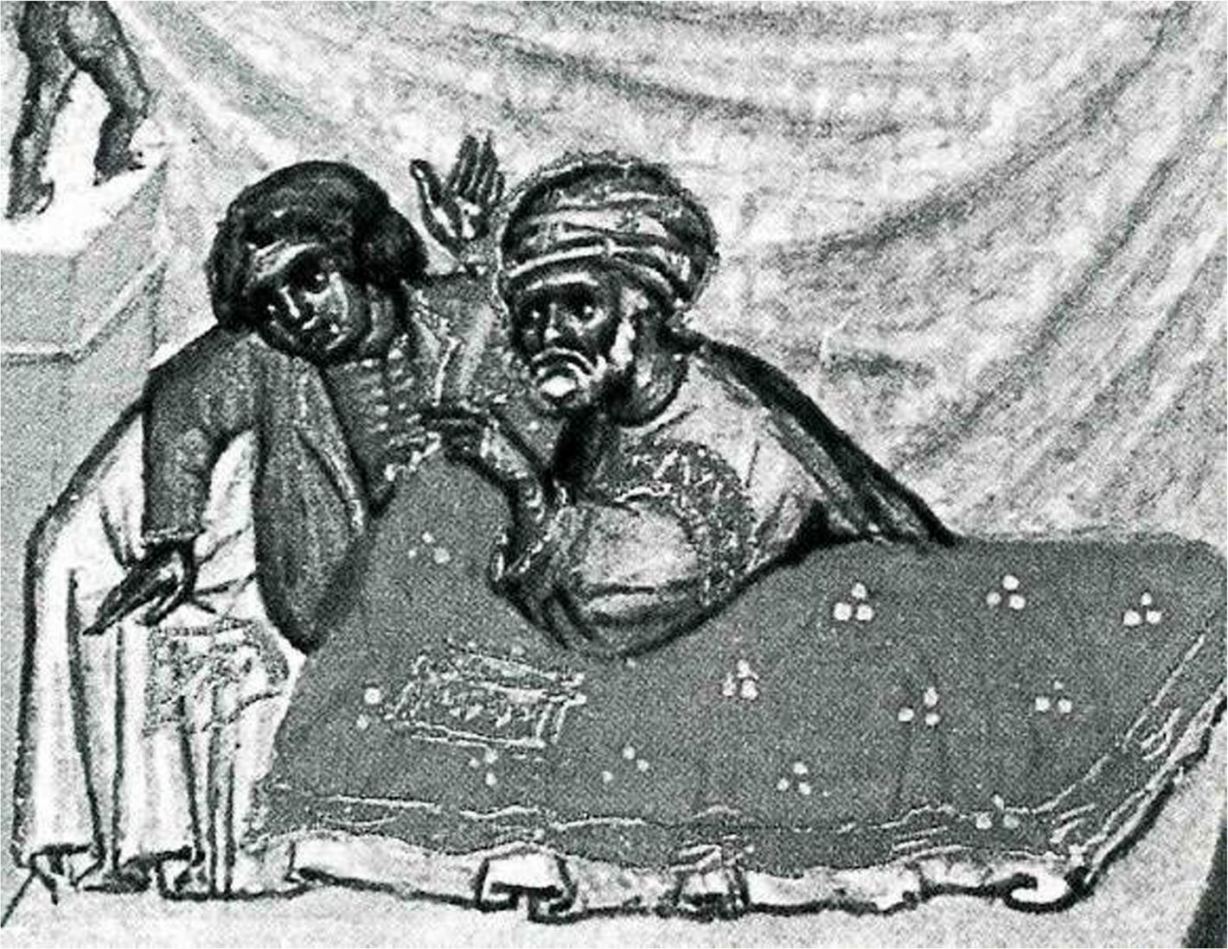
Treatment of chronic apoplexy. Miniature from ABU’L QĀSIM, Codex Series Nova 2641, Fol. 6ra. Reprinted in: (1979) Chirurgia. Lateinisch von Gerhard von Cremona. Vollständige Faksimile-Ausgabe im Originalformat. Graz: Akademische Druck- u. Verlagsanstalt. With permission of Austrian National Library, Vienna

The only pre-modern illustration depicting a stroke victim provides an excellent example for a “strange object” [Fig. 1]. The patient suffers from a so-called “chronic stroke”. Some very strange things are happening: the physician, kneeling on the floor behind the sickbed, holds a hot iron in his hand and starts to cauterize the patient’s stomach (or head?) with the glowing instrument. The patient is not unconscious and doesn’t seem to suffer from paralysis, since he is defending himself. To understand this scenery, our present-day knowledge of stroke is obviously not sufficient. In this case, the historian has to act like an ethnologist – looking not only at the medical, but also at the cultural context. All in all, however, the early story of stroke is taking place in scriptoria and scholars’ parlors, not at the bedside of hospitals or in labs. The study of its Western variant requires a solid knowledge of Ancient Greek and Latin. Additional reading ability in Arabic, Hebrew, Syriac, and other Semitic languages are quite helpful, too.

Assuming we are able to combine these two different models successfully – from which point of view do we want to look at the evolving drama?

- For doctors it seems clear that they are mainly interested in a medical perspective, in the reconstruction of changing medical knowledge. They will exclusively look at medical writings, perhaps also at medical illustrations, instruments, and institutions.
- But this is of course not the whole story. What about patients suffering from stroke? Granted they are somehow part of the parcel, we have to broaden our historical horizon and consult their diaries, autobiographies and other source material [6]. Not an easy task: There are few so called ego-documents from the pre-1800 period and we are dealing with a condition including aphasic and agraphic disturbances.
- How and where does society come into play? Every civilization is characterized by a spectrum of attitudes towards severe diseases of its members. Vice versa, severe diseases like stroke have a certain “image” generated within a social framework [7, 8]. To understand societal and financial aspects, historians may therefore want to resort to various other sources including balance sheets of hospitals, communities or ministries of health as well as the archives of the health industry. Finally, for assessing the public image of stroke, fictional literature [9, 10] as well as films, music and artwork constitute very important repositories.

**Fig. 2:**
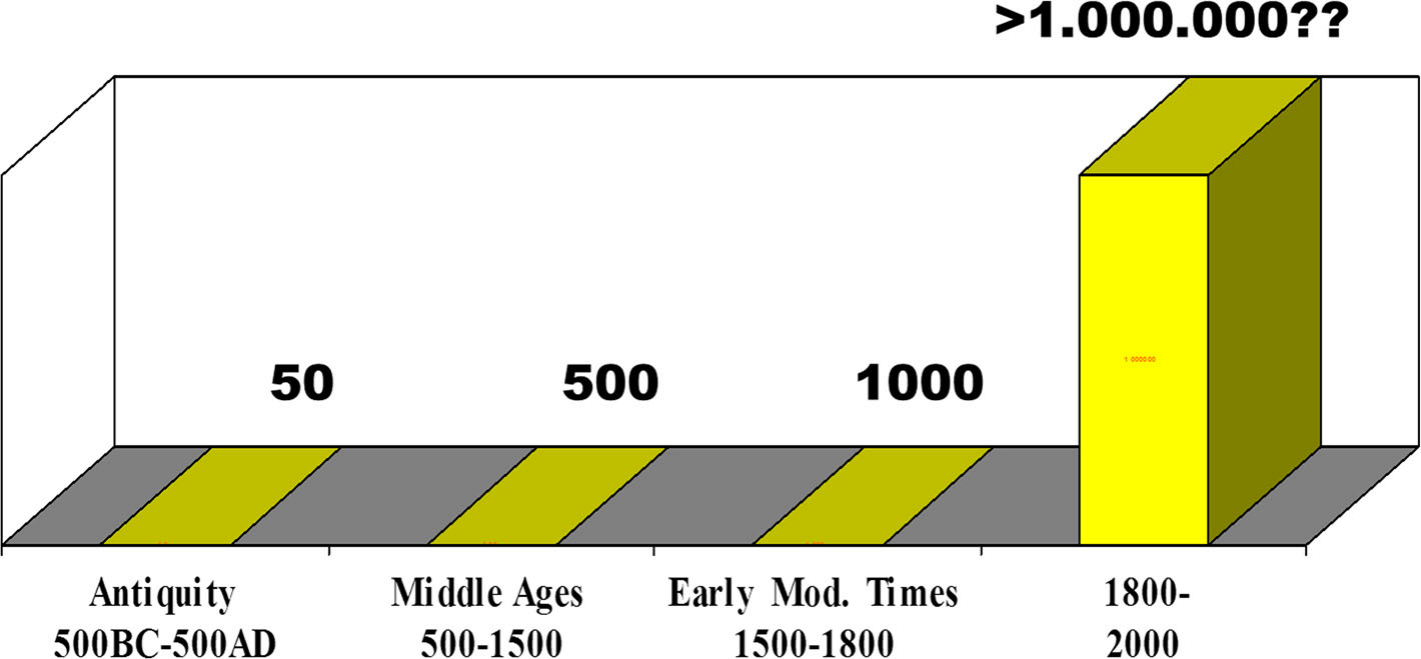
Estimated numbers of medical sources in the history of stroke across centuries. Drawing by the author

Why bother with all this theoretical stuff? It is important to understand that there will never be a single, all-encompassing history of stroke. There will only be various histories characterized by one or the other perspective and one or the other objective. This assertion is supported by a brief look at the number of available sources [Fig. 2]. Whereas the study of medical writings from antiquity, Middle Ages and Early Modern Times is manageable, no single historian will ever be able to read more than a minute part of the materials produced after 1800.

### Antiquity

An outline of the history of stroke usually begins with the Greco-Roman civilization [11–18]. It is, however, less conventional to follow the Socratic method based on the premise that one lived in a time of complete ignorance.

In antiquity, there were no dissections and no experiments besides some remarkable exceptions. The system of blood circulation was unknown, and regarding early antiquity no distinction could be made between arteries and veins. There was no consensus whether the brain or the heart was the instrumental organ for motor and sensory activities: whereas Plato, and most of the Hippocratic authors and Galen insisted on the brain [19], Aristotle and the physician-philosophers of the Stoic and Epicurean school favoured the heart [20, 21] – for good reasons, by the way.

Nevertheless, the ancients came up with many insights worth mentioning today. First, they produced descriptions of the disease such as: “Pain suddenly seizes the head in a healthy person, and he at once becomes speechless … and gapes with his mouth” [22]. In a further Hippocratic writing, one can read: “In apoplexy, drowsiness befalls this patient, he is senseless … mild fever is present, and his body is powerless. He dies on the third or fifth day, and generally does not reach the seventh” [23]. The authors of these statements did not use the word stroke, but apoplexy, which was the preferred medical term to label stroke-like conditions up to 1800 [24–26]. The explanation for its use is simple. Many early civilizations were convinced that acute diseases with loss of consciousness were sent by the gods. In Homeric Greek, some four centuries before Hippocratic physicians appeared on the scene, stroke meant “plex” or “plexy” and god meant “theos” or “dios”. Thus “theoplexy” or “dioplex” were common denominations for stroke, and apoplexy is nothing but a secularized version for a “very severe blow”. The definitions mentioned above and the etymology of the word make it clear that the ancient term “apoplexy” and its modern sequel “stroke” are all but equivalent. Apoplexy is an umbrella term under which one can easily summarize what we today call stroke, but one can also subsume myocardial infarction or pulmonary embolism triggering disturbances of consciousness.

Secondly, the Greeks realized that apoplexy was characterized by a different set of symptoms and a different course in comparison to various other medical conditions they called epilepsy, catalepsy, lethargy etc. [27]. Thirdly, ancient physicians wrote at length about treatment and prognostics including the world famous aphorism “It’s impossible to cure a violent attack of apoplexy, and difficult to cure a mild one” [28]. Finally, they set ethical standards how patients should be treated which did not change very much until today in many parts of the world.

But how did Greco-Roman physicians explain the disease? Now it’s time to remember the “strange object approach”. Galen, a Greek physician practising and writing around the year 200 of our era in Rome, developed the idea that a vital spirit built in the heart was carried towards the brain via the arteries. In a structure called retiform plexus (which he found in several animal species in the region around the pituitary gland) this vital spirit was then transformed into the so-called animal spirit. Only the animal spirit was stored in the cerebral ventricles from where it acted on demand as a “fuel” for the transmission of motor impulses and sensory data to the periphery. That’s, in a nutshell, how the brain worked according to Galen [29].

**Fig. 3:**
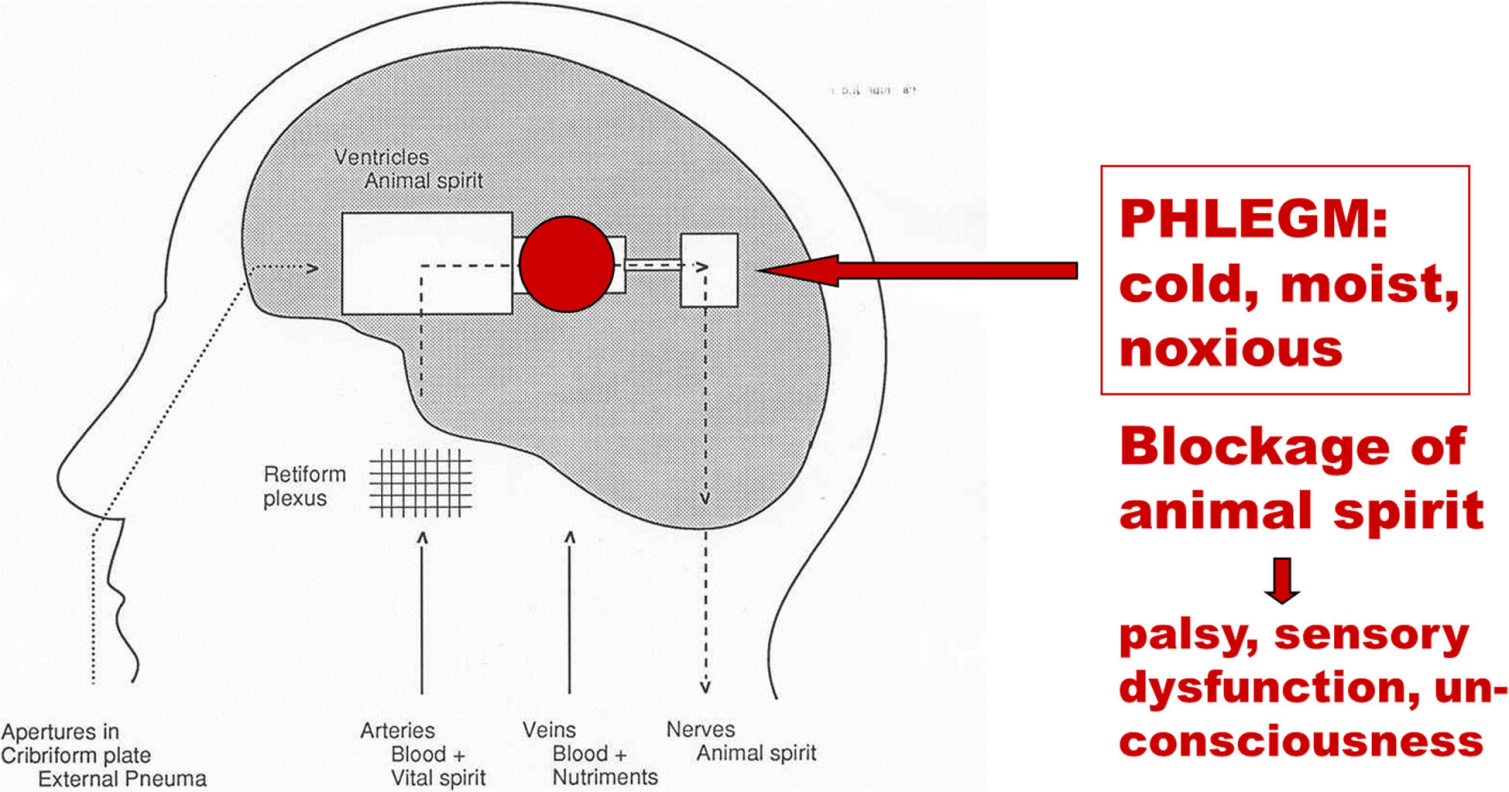
Galen’s doctrine of apoplexy. Drawing by the author

In individuals with apoplexy, a noxious humour called phlegm accumulated in the body: “When the vessels drew phlegm into themselves, the blood must, on account of the coldness of the phlegm, stand more still than before and be cooled, and so, with the blood immobile, it is impossible for the body not to become still and numb … But if the phlegm predominates, the blood is cooled and congeals more, and if it reaches a certain stage of cooling and congelation, it congeals completely, the person becomes cold, and he dies” [22]. The accumulation of noxious mucus thus blocked the flow of the animal spirit, and the blockage eventually resulted in palsies, sensory dysfunctions, loss of consciousness, and possibly death [Fig. 3]. Phlegm was a cold, moist and viscous fluid which could accumulate in the brain when the body was exposed to too much cold, e.g. during the winter, in old age etc. [30]. This theory was the fundamental etiological doctrine of stroke and other brain diseases which dominated European thinking for 1500 years. No text on brain disorders up to 1750 can be understood without a solid knowledge of these principles.

This doctrine was also the basis for various therapeutic actions. A practising physician and adherent of this teaching had two basic options: either evacuation – meaning “cleansing brain and body” in order to get rid of the superfluous fluid, or counter-action – i. e. applying something hot and dry to combat the cold and moist humour. Evacuation and counter-action are two of the major meta-strategies of medical treatment until today. If one adds the four major realms of pre-1800 treatment – physical therapy, dietetics, herbal pharmacology and surgery – a wide spectrum of therapeutic measures is available, ranging from hot bathing to various surgical procedures including cauterization [31].

So what we see in ancient times is a rational, but speculative explanation of symptoms. This explanation was only loosely tied to the observation of nature, focused on “hidden causes” of disease but deeply embedded in ancient philosophy. Harmony between man and world, and harmony between the various parts of the body were its key principles. It is this holistic approach which makes certain aspects of ancient medicine still attractive for a couple of contemporary physicians representing alternative forms of medicine [19].

### The medieval period

**Fig. 4:**
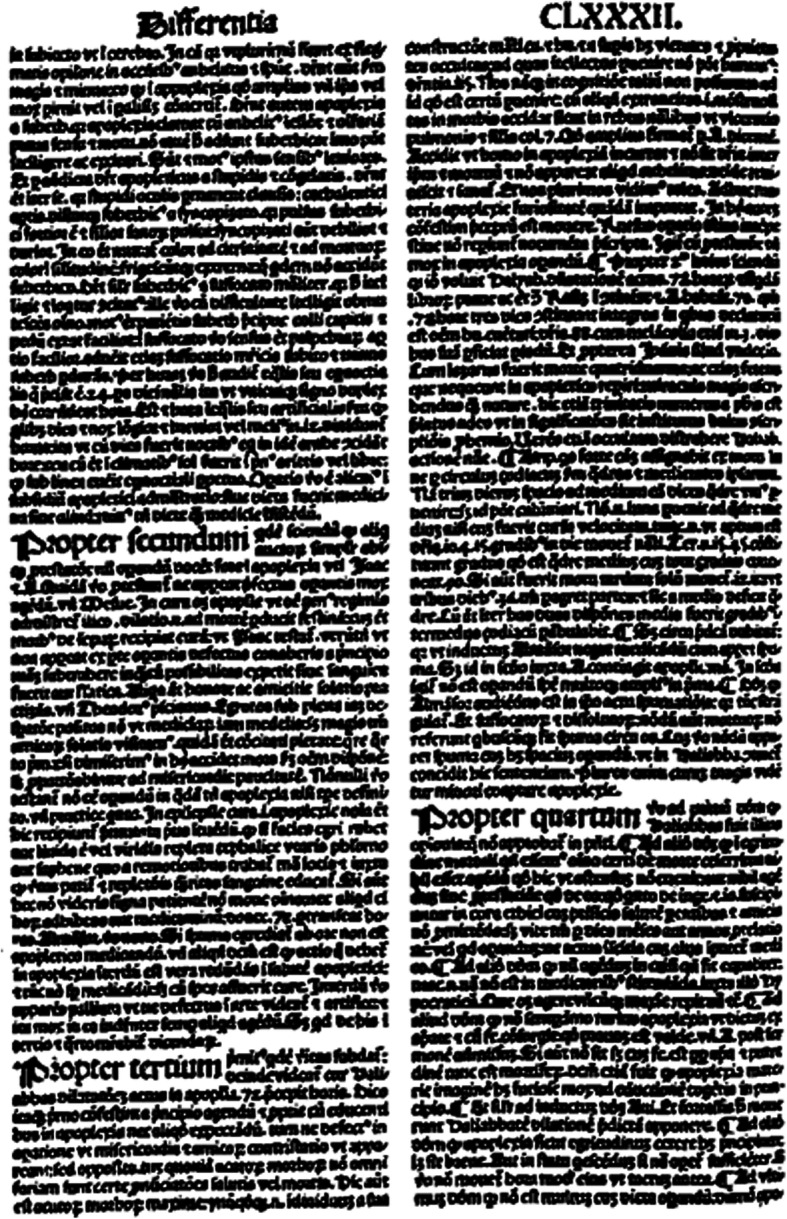
Folio page from Pietro d’Abano’s Conciliator (1496). With permission of the University and City Library of Cologne

Between 500 and 1500 Galen’s brain-centred doctrine of neuropsychiatric symptoms was important, but it was, as mentioned before, not the only one. Aristotle and his followers had propagated a powerful cardiocentric theory: a teaching based on the assumption that the origins of motion, sensation and consciousness were located in the heart. “Tell me where is fancy bread, or in the heart or in the head”: with regard to stroke this question formulated by William Shakespeare centuries later was the neurological hotspot around the year 1000 [32]. Especially the best physician-philosophers of the rising Islamic civilization tried to reconcile both doctrines. Rhazes and Avicenna (ar-Razi and ibn-Sina) felt that Aristotle as well as Galen had good arguments [33]. From the eleventh century on, the issue was transferred from Bagdad and Cairo to the Latin West, to the emerging centres of learning in Italy, Spain, France, England, and finally the German speaking territories. Since there were still no dissections or experiments at hand, the problem couldn’t be solved in a modern way. But it was solved in a medieval way: by playing with words and quoting authorities. There are about a dozen truly scholastic texts on stroke, full of arguments and counter-arguments, pseudo-problems and pseudo-answers [32]. A folio page of a writing by the Italian physician-philosopher Pietro d’Abano [Fig. 4] [34, 35], who taught in Padova in the early fourteenth century, provides insight into these sophisticated discussions and their fruitlessness from the patient’s point of view. By the way: the final medieval solution of the brain-heart-issue was to emphasize that a stroke began in the brain but terminated in the heart. This solution was accepted until the seventeenth century.

Another “highlight” of the medieval era is the occurrence of miracles and patron saints. Early examples of faith healing in stroke and other medical conditions can be found in the Byzantine Empire, later ones in the Latin West and the Orthodox church of the East. These documents are ranking among the most important sources that have come down to us from these centuries. To quote just one miracle story which took place in Italy around the year 1250 in a Franciscan monastery, about one generation after St. Francis died:

“A certain young man … was subjected to a terrible fright that provoked mental confusion and paralysis of the right side of the body. Through his severe illness, he also lost hearing and the movement and sensation of the tongue. He had been confined to bed for some day in this pitiful state … One morning St. Francis appeared in the infirmary … stretched out his hand, running it lightly over the novice’s right side from head to foot, touching him gently, and placed his fingers in the young man’s ears, saying: ‘This shall be for you a sign that God has through me … completely restored your health’. With these words … the young man arose and entered the church with his health of body and mind, to the great astonishment of the brothers” [36].

This “case report” can be analysed in various ways [37]. A contemporary neurologist may think of a reversible ischemic neurological deficit or a psychogenic condition. A historian will certainly look for the theological and literary context. It is all but easy to arrive at a satisfactory conclusion here, also because this trove of miracle cures is largely unexplored yet.

A closing remark about stroke and religious healing in the Middle Ages. For epileptics, there were more than a dozen patron saints which could be addressed for prevention and/or cure of the illness [38]. For apoplectics, there was none. St. Wolfgang and St. Andreas Avellino only had a minor local significance [39]. For the scholar studying medieval medicine this shortage is quite surprising, and of course such a negative result needs an explanation. It certainly has to do with the high fatality rate of stroke: Patron Saints are no emergency room. But it may also be linked to the high rate of post-stroke disabilities hindering victims to travel to a place of pilgrimage. On the other hand, there was definitely a lack of commercial interest on the part of these places of pilgrimage – one of the first “management mechanisms” in the care of stroke patients.

### 1500–1750

Books and chapters about medicine in the Renaissance often use the metaphor of a “wind of change”, alluding to a fresh breeze blowing constantly over the European continent and sweeping away the medieval dust. But is that really true? As far as the history of stroke is concerned: yes, and no.

Following the first anatomical dissections of human bodies around 1300, from 1450 onward an increasing number of post-mortem examinations were carried out, leading to a better knowledge of brain anatomy and brain vasculature. About a century later, the first autopsies were performed in order to discover bodily changes in certain diseases: pathological anatomy was born. And in 1628 William Harvey used a variety of sophisticated experiments to prove that there is really something like a circulation of the blood, a circulation through the whole body including the head and the brain. New methods, revolutionary findings: but their significance for the understanding of stroke was practically zero, at least at the beginning.

In 1602, the Swiss physician Felix Platter carried out a brain autopsy after the death of one of his stroke patients. He summarized his findings of the post-mortem with the following words:“ a phlegmatic humour is obstructing the inner passages of the brain” [40]. This short statement highlights two pivotal insights that the study of historical diseases provides. First: every scientific observation is theory-laden, then and now. Second: obviously it is very difficult to get rid of traditional beliefs. Platter and practically all of his colleagues [41–44] subscribed to Galen’s age-old idea that apoplexy was caused by phlegm in the cerebral ventricles. And what did brain autopsy reveal: phlegm in the cerebral cavities.

Yet brain autopsies plus the idea of a circulation marked the end of the pre-modern era of stroke. A true figure of transition was another Swiss physician named.

**Fig. 5:**
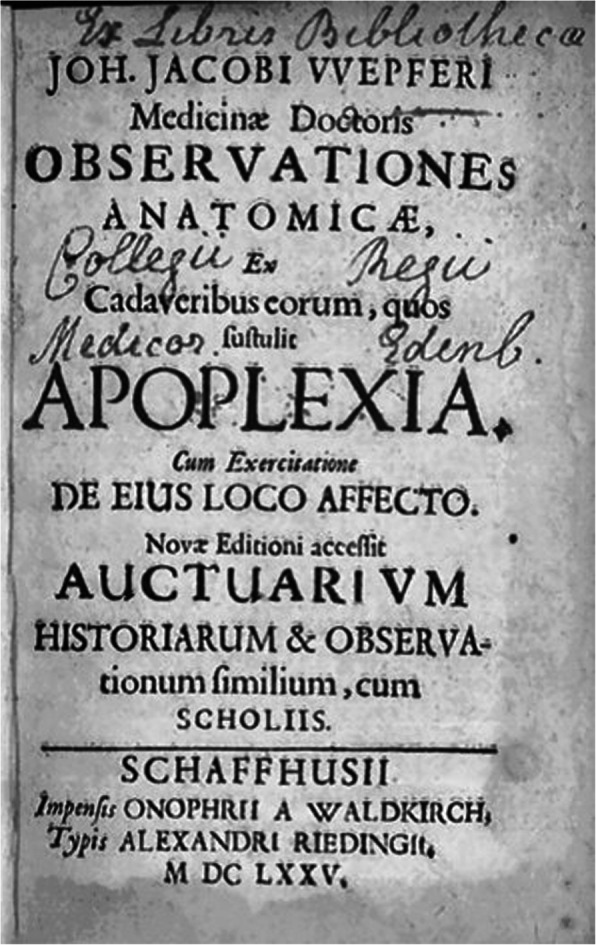
Title page of Wepfer’s 1658 monograph on stroke. New edition 1675. With permission of the Institute for the History of Medicine and Medical Ethics, University of Cologne

Johann Jakob Wepfer, who authored one of the most famous monographs on stroke of all times [Fig. 5]. For a long time, clinicians tended to classify Wepfer as a modernist and true reformer, for a couple of good reasons. With his research apoplexy became a cerebrovascular disorder. Moreover, he was convinced that the pathological changes were located in the cerebral substance and not in the ventricles. He also gave the hitherto most precise description of the encephalic arteries including the circle of Willis. In this context, Wepfer discovered during his dissections “pituitous formations” in brain arteries and hypothesized that these formations (clots?) play an important role in stroke. In addition, he found a hemorrhage in about 50% of the brains of stroke victims, thus proving that the rupture of a cerebral artery is responsible for the subsequent attack [45]. Wepfer was therefore praised as one of the harbingers of modern medicine [46–49]. However, a careful reading of his 400-pages book [50–52] – a tedious task, because it is written in a difficult Latin – leads to striking results. Wepfer was a modernist, but at the same time still also a true follower of Galen with more than old-fashioned ideas on cerebral physiology and the etiology of stroke. According to him, the obstruction of brain arteries was harmful because the little clots blocked the flow of the vital spirit from the heart to the brain. A hemorrhage in the brain was disastrous because the animal spirit couldn’t flow freely towards the spinal cord and the nerves, and so on. Basically, Wepfer’s concept of stroke was still an ancient one dealing with humours and spirits, only supplemented by the latest anatomical and pathoanatomical discoveries.

### The modern era

The decisive difference between the pre-1800 and the modern period in the history of stroke is therefore not to be found in a single discovery or a set of new methods, but in a change of the basic notion of what a disease is [53]. From Galen to Wepfer, stroke (like many other neurological conditions) was defined as a collection of certain symptoms. With the beginning of the nineteenth century, however, stroke is for the first time defined as a result of a lesion. The morphological lesion, and only the morphological lesion, became the decisive criterion for an operational definition of stroke; the symptoms were now only seen as “indicatory signs”. Henceforth morbid anatomy, and nothing but morbid anatomy, became the key basis of all knowledge about stroke.

**Fig. 6:**
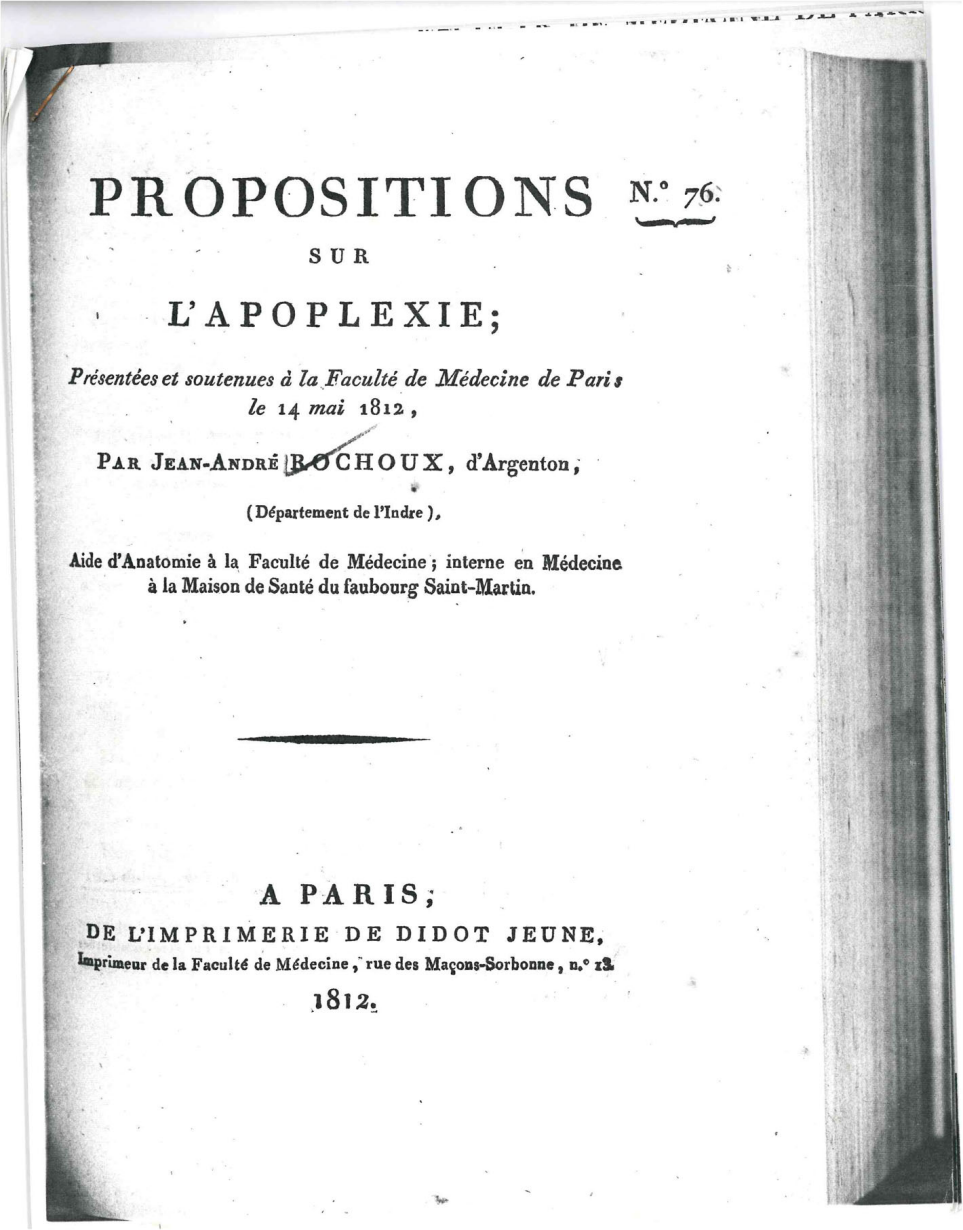
Title page of Rochoux’ 1812 dissertation on cerebral hemorrhage. With permission of the Institute for the History of Medicine and Medical Ethics, University of Cologne

The birth of this new way of perceiving a disease is inseparably linked to the Paris school of medicine. Therefore, it is no surprise that the first truly modern definition of stroke is to be found in a French publication. In 1812, the young physician Jean-André Rochoux [54] produced what can be called the most important dissertation in the history of neurology [Fig. 6]. His text started with a phrase that is a platitude today, but indicated a scientific revolution when it was printed: “Apoplexy is a hemorrhage of the brain, by rupture, with more or less serious alteration of its substance” [55]. First Rochoux explained the lesion, its size, colour and location, then traced the signs and course of stroke, and concluded with a few remarks on treatment and prognosis. Undoubtedly, his dissertation and the monograph he produced 2 years later [56] are the first modern texts on stroke.

But Rochoux’ thorough exposition remained not the only one. In the very same decade, another young French doctor by the name of Léon Rostan came up with the idea that stroke must be the result of a softening of the brain (“ramollissement du cerveau”) [57]. In modern terms: ischemic infarction of the brain. Thus by 1820, the two basic modern concepts of stroke had been discovered. They were both offspring from the anatomico-clinical method, i. e. the comparison of post-mortem lesions with in-vivo symptoms. They were both found through hospital-based research and grounded on statistical observations. What followed during the whole 19th and a good part of the twentieth century was nothing but a refinement of these two basic concepts. Therefore, one can now really talk about progress and milestones: for example, the discoveries made by Rudolf Virchow who described, starting in 1846, arterial thrombosis and embolism and recognized the interaction between blood and arterial wall. Furthermore, the Berlin pathologist clearly showed that vascular occlusions caused infarction [58]. Again, this is a triviality today, but was a matter of acrimonious scientific debates in the 1840s.

From the mid-nineteenth century onwards, medicine as a branch of knowledge was inextricably linked to the natural sciences. “No other theory as that of facts” was the positivistic credo of the epoch. A prominent German clinician put it even more bluntly: “Medicine will either be science or cease to exist”. Research on stroke profitted enormously from this positivistic movement. An increasing number of physicians, some of them specialized in neurology, contributed to the rapidly growing treasure of knowledge [59–62]. It is impossible to enumerate all the important contributions, but one can at least emphasize some historical trends. From 1850 to 1930, vascular anatomy and clinical-anatomical correlations were of special interest, including various brain stem lesions. Between the 1920s and the 1970s, the pathophysiology of vascular lesions reached its hey-day including figures like the Frenchman Charles Foix, Kapitoline Wolkoff from St. Petersburg, and the Canadian Charles Miller-Fisher [13].

From about 1975 onwards, research on risk factors, stroke registries, randomized trials, databases, as well as a general momentum for “new treatments” can be observed [63]. So by the end of the twentieth century, stroke medicine became a subspecialty of neurology, theoretically as well as practically. And another important fact should be emphasized: For the first time, treatment of stroke was characterized by what one can call “limited therapeutic optimism” [64]. To a large extent, however, this evolution was due to the technical development of diagnostics: the history of twentieth-century medicine is essentially a history of technology. In the case of neurology, new technologies were available almost every decade, from hands-on spinal tap around 1900 to the most sophisticated brain visualizations 100 years later [65]. Sometimes it was even difficult for the doctor’s brains to keep up with the speed of technical innovation. But what is certain is that the history of stroke in the twentieth century remains to be written.

## Conclusions

What are the take home messages of this historical tour?

- It is a matter of personal taste if one likes the allegory of the dwarfs standing on the shoulders of giants. However, knowledge about the history of one’s own field almost automatically leads to professional and personal modesty, which is always a good attitude, especially for physicians.
- An intellectual tour through history may raise the awareness of how intimately we are connected to the intellectual, technical, and social context of our own epoch – we may or may not realize this frame. Galen, Wepfer, Rochoux, Rostan, Virchow and all the other important figures in the history of stroke: they were all products of their times sharing certain opportunities and certain limits – and so are we.
- Perhaps the most valuable insight gained is that “unthinking” ideas of the past can be a key to great scientific success. The modern era in the history of stroke only came true because physicians of the early nineteenth century “unthought” all the misleading and constrictive presumptions which had been around for centuries. A fundamental conclusion therefore is: You can change history, yes you can!

This essay shall be concluded with an encouraging quote from the French poet André Gide expressing the very same idea in a much more elegant way:

“One does not discover new continents without consenting to lose sight of the shore for a very long time” [66].

## Data Availability

Not applicable.
